# Genomic comparison of closely related Giant Viruses supports an accordion-like model of evolution

**DOI:** 10.3389/fmicb.2015.00593

**Published:** 2015-06-16

**Authors:** Jonathan Filée

**Affiliations:** Laboratoire Evolution, Génome, Comportement, Ecologie, Centre National de la Recherche Scientifique UMR 9191, IRD UMR 247, Université Paris-SaclayGif-sur-Yvette, France

**Keywords:** giant virus, genome, lateral gene transfer, duplication, evolution, mobile genetic elements

## Abstract

Genome gigantism occurs so far in Phycodnaviridae and Mimiviridae (order Megavirales). Origin and evolution of these Giant Viruses (GVs) remain open questions. Interestingly, availability of a collection of closely related GV genomes enabling genomic comparisons offer the opportunity to better understand the different evolutionary forces acting on these genomes. Whole genome alignment for five groups of viruses belonging to the Mimiviridae and Phycodnaviridae families show that there is no trend of genome expansion or general tendency of genome contraction. Instead, GV genomes accumulated genomic mutations over the time with gene gains compensating the different losses. In addition, each lineage displays specific patterns of genome evolution. Mimiviridae (megaviruses and mimiviruses) and *Chlorella* Phycodnaviruses evolved mainly by duplications and losses of genes belonging to large paralogous families (including movements of diverse mobiles genetic elements), whereas *Micromonas* and *Ostreococcus* Phycodnaviruses derive most of their genetic novelties thought lateral gene transfers. Taken together, these data support an accordion-like model of evolution in which GV genomes have undergone successive steps of gene gain and gene loss, accrediting the hypothesis that genome gigantism appears early, before the diversification of the different GV lineages.

## Introduction

The order Megavirales (formerly NCLDVs: Nucleo Cytoplasmic Large DNA Viruses) comprises very diverse double strand DNA viruses infecting a wide range of eukaryotic hosts, including vertebrates, protists, and unicellular alga (Colson et al., 2013). This group is supposed to be monophyletic on the basis of a conserved set of 25–50 genes encoding mainly structural and informational proteins (Iyer et al., 2006; Yutin et al., 2013). Seven different families compose the order: Poxviridae, Asfarviridae, Iridoviridae, Ascoviridae, Phycodnaviridae, Mimiviridae, and Marseilleviridae. Among them, two families display important variations in term of genome size: the Mimiviridae ranging from 371 to 1259 kb (Arslan et al., 2011; Moniruzzaman et al., 2014) and the Phycodnaviridae ranging from 160 to 2550 kb (Lee et al., 1995; Philippe et al., 2013; Yutin and Koonin, 2013). Viral genome gigantism challenges the traditional definition of viruses conceived as small and simple organisms. Understanding the evolutionary forces acting on the Giant Virus (GV) genomes have profound implication for our knowledge of the viral world and the interplays between cellular organisms and viruses. Additionally, there are accumulating evidences that several GVs caused respiratory tract infections. Although the exact mechanism of their pathogenocity is currently unknown, mimiviruses are suspected to be the ethiologic agents of numerous cases of pneumonia acquired by patients in intensive-care institution but also by apparent healthy patients (Kutikhin et al., 2014). Origin of genome gigantism in these families is still a matter of an intense controversy between the advocates of the “genome degradation hypothesis” (Claverie, 2006) and those defending the “genome expansion hypothesis” (Moreira and Lopez-Garcia, 2005; Filee et al., 2008; Yutin et al., 2014). The “genome degradation hypothesis” postulates that Giant Viruses (GVs) derive from a cellular ancestor by progressive genome simplification linked to the adaptation to a parasitic lifestyle. Notably, presence of typical cellular hallmark genes as translational genes, supports the hypothesis that GVs derive from a cellular ancestor (Arslan et al., 2011). However, phylogenetic studies indicate that most, if not all, of these translational genes result from lateral gene transfers (LGTs) from cellular organisms (Moreira and Lopez-Garcia, 2005; Yutin et al., 2014). Indeed, many studies have pointed out the central role of lateral gene transfers during the evolution of GVs (Iyer et al., 2006; Filee et al., 2007, 2008; Moreira and Brochier-Armanet, 2008; Yutin et al., 2013). Finally, gene and genome duplications (Suhre, 2005; Filee and Chandler, 2008) in addition to dissemination of various mobile genetic elements as introns or transposons (Filee et al., 2007; Desnues et al., 2012) have also been identified as important player for GV genome evolution. The combination of these forces support the “genome expansion hypothesis” in which GVs evolved from a relatively simple viral ancestor by progressive gene accretion and duplication. The nature of this ancestor remains speculative but recent discoveries indicate that GVs may derive from DNA transposons belonging to the Polinton/Virophage superfamily (Krupovic and Koonin, 2015). However, experimental data have shown that under laboratory conditions, members of the Mimiviridae can experience rapid genome expansion/contraction. Indeed, under peculiar selective constraints, Poxvirus genomes undergo successive steps of gene duplications and gene losses (Elde et al., 2012). Symmetrically, when mimiviruses are cultivated in bacteria-free media (the preys of their amoebal hosts), numerous genome reductions occur, mainly caused by large deletions (Boyer et al., 2011). On the basis of these data, I recently proposed a model in which GVs evolved using a complex process of “genomic accordion” instead of a general tendency toward either genome expansion or reduction (Filee, 2013). According to this hypothesis, GVs should undergo successive cycles of genome expansion and reduction in order to adapt to modified environmental conditions or new hosts. This hypothesis is readily testable by genomic comparisons of closely related GVs. Thus, I took advantage of the availability of clusters of very similar GVs genomes belonging to the Phycodnaviridae and the Mimiviridae families to analyze the pattern of gene loss/gene gain, using whole genome alignments. In this paper, I show that genomic mutations occur regularly over the time (clock-like). However, a global trend of genome evolution was not observed, each lineage having a specific mode of evolution as duplication and subsequent loss of genes belonging to large multigene families (mimiviruses and megaviruses), recursive insertion and excision of transposons and mobile endonucleases (*Chlorella* viruses) and acquisition of new genes via LGTs (*Ostreococcus* and *Micromonas* viruses). Thus, these observations support the hypothesis that ancestors of the present-day GVs were already GVs that have evolved through a balanced, predominantly neutral process of gene gains and gene losses.

## Material and methods

### Data collection

GV genomes were downloaded from the NCBI Viral Genome database at http://www.ncbi.nlm.nih.gov/genome/viruses/. Minimal requirements needed are: at least three nearly complete genomes per group with sufficient genome syntenies to identify orthologous genes without ambiguities. As GVs display numerous gene duplications (Suhre, 2005), synteny is a determinant criterion to recognize the orthologous genes by positional homology. Identical or nearly identical genomes as the three *Phaeocystis* viruses for example were excluded. The final dataset includes 4 mimiviruses and 4 megaviruses for the Mimiviridae family and 3 *Micromonas* viruses, 5 *Ostreococcus* viruses, and 5 *Chlorella* viruses for the Phycodnaviridae family (Table 1).

**Table 1:** **Major characteritics of GV genomes and description of the genomic events that have occured after the divergence with their respective common ancestors**.

**Virus name**	**Genbank**	**% Identity ancestor**	**Size (kb)**	**Duplication**	**LGT**	**Insertion**	**Total Gain**	**Loss**	**Excision**	**Total loss**	**Unknow (Orphan)**	**Translocation**	**Genomic Events**
Mimivirus	NC_014649.1	99	1182	5	0	1	6	7	0	7	0	0	13
Mamavirus	JF801956.1	99	1192	5	0	0	5	7	0	7	3	8	23
Hirudovirus	KF493731	99	1181	3	0	1	4	6	0	6	0	1	11
Terra2	NC_023639	99	1169	10	3	2	15	19	1	20	3	3	41
Megavirus Chiliensis	NC_016072.1	98	1259	9	1	2	11	5	0	5	4(0)	0	20
Megavirus lba	NC_020232	97	1231	9	0	1	10	24	0	24	4(0)	1	39
Megavirus courdo11	JX975216	98	1246	6	1	3	10	15	1	16	10(2)	0	36
Megavirus terra1	NC_023640	99	1245	12	0	1	13	8	1	9	1(0)	0	23
OtV1	NC_013288	88	192	3	5	0	8	9	0	9	4(1)	0	21
OtV2	NC_014789.1	87	184	1	13	0	14	12	0	12	7(4)	0	33
OsV5	NC_010191	89	185	1	5	0	6	4	0	4	7(4)	0	17
OlV1	NC_014766	85	194	1	11	0	12	8	0	8	4(2)	0	24
OtVRT-2011	JN225873	92	190	0	12	0	12	7	0	7	18(9)	0	37
MpV SP1	JF974320	82	173	1	8	0	9	20	0	20	14(3)	0	43
MpV1	NC_014767	83	184	0	8	0	8	16	0	16	27(16)	0	51
MpV PL1	HQ633072	81	197	12	9	0	21	17	0	17	23(15)	0	61
Nys-1	JX997183	98	348	3	0	3	6	13	2	15	6(1)	0	27
NY2A	NC_009898	94	369	16	4	6	26	14	1	15	7(5)	0	48
AR158	NC_009899	99	345	7	3	2	12	9	4	13	2(1)	0	27
IL-5-2s1	JX997170	99	345	1	0	1	2	3	3	6	3(0)	0	11
MA-1D	JX997172	99	340	0	0	2	2	9	6	15	2(1)	0	20
				105	83	25	212	232	19	251	150(74)	13	626

### Whole genome alignment

In order to identify positional homologs and infer the ancestral state of each gene, multiple genome alignments were carried out and visualized using progressiveMauve (Darling et al., 2004). Whole genome phylogenetic trees based on gene content/substitution distances were computed. Roots of trees were chosen to minimize the evolutionary distance: i.g. the mimivirus tree was rooted with a megavirus and vice versa. Concerning the *Chlorella* viruses, as the *Ostreococcus* and the *Micromonas* virus genomes are too divergent to find reliable genome syntenies, the sequence of *Chlorella* virus PBCV-1 genome was used. Two by two, whole genome comparisons were achieved with wgVISTA (Couronne et al., 2003) and unaligned DNA segments larger than 300 bp containing at least one ORF were extracted. These sequences were searched with a TBLASTN (Altschul et al., 1990) against a NR database to identify the genomic variations (gene transfers, gene duplications, gene losses, movement of mobile genetic elements, orphan etc…). In order to identify the nature of the genomic events (a gene duplication rather than a gene loss for example), the most parsimonious scenario principle was applied using the whole genome trees as templates. When two scenario are equally parsimonious (implying the same number of mutation steps) the corresponding genes were marked as “ambiguous.”

### Sequence analysis and phylogenies

Four taxonomic markers (DNA polymerase, A2L Transcripition factor, D5 Primase-helicase, packaging ATPase) were used to build a global phylogenetic tree of the GVs used in this study. The ancestral sequence of each group was reconstructed using FastML (Ashkenazy et al., 2012) and pairwise comparisons were computed using BLASTN (Altschul et al., 1990).

Sequences were aligned using MUSCLE (Edgar, 2004) with default parameters and conserved parts of the alignments usable for phylogenetic analyses were chosen using Gblocks (Castresana, 2000). Concerning phylogenies, best-fitting ML models were selected using Protest (Abascal et al., 2005) and the trees were implemented using PhyML 3.0 (Guindon et al., 2009). Branch supports were calculated using a LRT Shimodaira-Hasegawa (SH) procedure.

Raw data including whole genome alignments, gene sequence alignments, reconstructed ancestral sequences, phylogenetic trees etc. are available at http://echange.legs.cnrs-gif.fr:5000/fbsharing/b95jcnuZ.

## Results

### A predominant model of neutral evolution due to regularly accumulated mutations over the time

The pattern of gene loss/gene gain at a small evolutionary scale for five groups of closely related GVs belonging to the families *Megavirales* and *Phycodnaviridae* was analyzed. Up to 626 genomic events were detected (Table 1) corresponding to 212 gene gains, 251 gene losses, and 163 ambiguous variants (including 74 orphans). Insertion and excision of mobile genetic elements are additional factors (44) and very few gene translocations have been evidenced (13). Gene duplication (105 events), lateral gene transfers (85) and gene losses (232) were by far the major evolutionary forces shaping the GV genomes.

**Figure 1:**
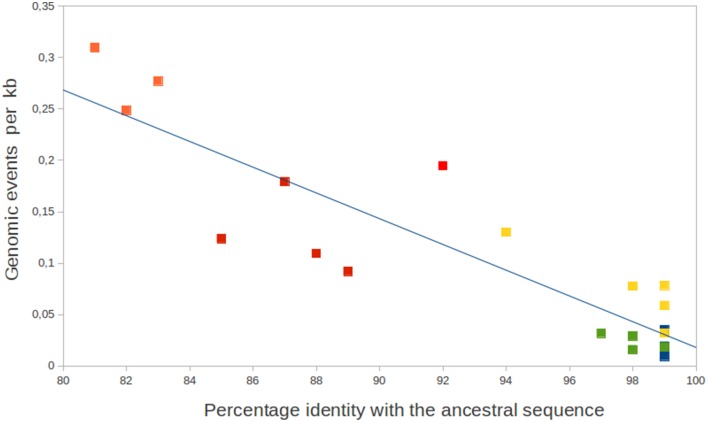
**Number of genomic events per kb for each GVs with respect to the percentage of nucleotide similarities of the four taxonomic genes with the ancestral sequences**. GVs are represented by squares: orange for *Micromonas* viruses, red for *Ostreococcus* viruses, green for *Chlorella* viruses, yellow for megaviruses, and blue for mimiviruses. The blue line represents the regression (*r*^2^ = 0.81).

For each group of GVs, ancestral sequence reconstruction of four taxonomic gene markers was computed. Percentages of identity between these ancestral sequences and the present ones were indicated in Table 1. If a molecular clock of sequence evolution is assumed, this analysis provides inside each group of viruses, a proxy of the time of divergence between each viruses and the last common ancestor of their respective groups. Thus, mimiviruses, megaviruses, and *Chlorella* viruses appear closely related (94 to 99% of sequence identity compared to their respective ancestral sequences) whereas *Ostreococcus* and *Micromonas* viruses are substantially more divergent (81 to 92% of sequence identity). Figure 1 plots the number of genomic events per kb for each virus, with respect to the sequence identity of the four marker genes. The regression line indicated that there is a negative correlation between the quantity of genomic variations and the sequence identity. In other words, the amount of genomic variations increased linearly with the time of divergence from the last common ancestor. There is no evident deviation from the regression line indicating slow down or increase of the rate of genomic mutation for peculiar groups or individual virus. Consequently, these data support the idea that GV genomic variations accumulate regularly over the time in an approximately clock-like manner. Adaptive genomic mutations could occur at a constant rate but a predominant neutral model of genomic evolution seems more possible.

### Evidences for group-specific evolutionary patterns

**Figure 2:**
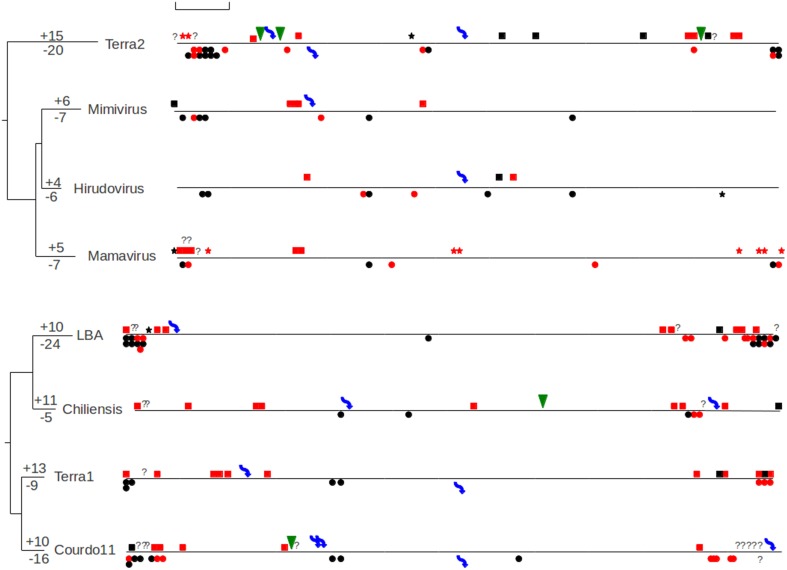
**Genomic map of the megaviruses and the mimiviruses describing the different genomic mutations that have occurred after the divergence with their last common ancestor genome**. Symbols below the line indicated gene losses (dots for gene losses, arrows for excision of mobile genetic elements and stars for translocation) and symbols above the line indicate gene gains (squares for duplications, triangle for LGTs and arrows for insertion of mobile genetic elements). Interrogation marks represent ambiguous cases or presence of orphans. Squares and dots in red indicate genomic events of genes belonging to paralogous families. The trees represent the whole genome phylogeny, the numbers above and below the terminal branches indicate the amount of gene gain and loss for each virus. The scale bar represents the equivalent of 100 kb.

**Figure 3:**
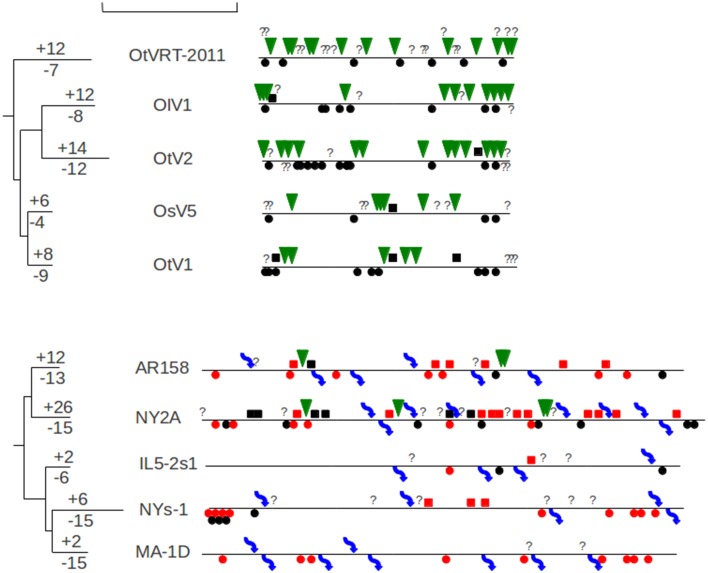
**Genomic map of the ***Chlorella*** and ***Ostreococcus*** Phycodnaviruses describing the different genomic mutations that have occurred after the divergence with their last common ancestor**. Symbols below the line indicated gene losses (dots for gene losses, arrows for excision of mobile genetic elements) and symbols above the line indicate gene gains (squares for duplications, triangle for LGTs and arrows for insertion of mobile genetic elements). Interrogation marks represent ambiguous cases or presence of orphans. Squares and dots in red indicate genomic events of genes belonging to paralogous families. The trees represent the whole genome phylogeny, the numbers above and below the terminal branches indicate the amount of gene gain and loss for each virus. The scale bar represents the equivalent of 100 kb.

Analyses of the spatial patterns of gene loss and gain along the genomes were displayed in Figure 2 (mimiviruses and megaviruses) and Figure 3 (*Ostreococcus* and *Chlorella* viruses). With the exception of the megavirus LBA, most of mimiviruses and megaviruses display an equilibrium between gains and losses. The megavirus LBA has significant higher number of gene losses than gene gains leading to a smaller genome compared to the other megaviruses (Table 1). As previously observed, these results confirm that the extremities of the *Mimiviridae* genomes are hot spots of genome variation (Filee et al., 2007; Colson et al., 2011). In addition, most of the genomic variations in the two viral groups concern multigenic families: more than 75% of duplications concern families with at least three additional representatives in the corresponding genomes and 50% of the gene losses also impact duplicated families. Duplicated genes families represent roughly one third of the total genes content of the mimivirus (Suhre, 2005) and of the megavirus *chiliensis* (Arslan et al., 2011). This over-representation of duplicated families compared to their current distribution in the Mimiviridae genomes suggests that they are the primary source of variation in this viral group. Indeed, LGTs, movement of genes or mobile genetic elements as transposon or mobile endonuclease and generation of orphans represent an anecdotal fraction of the genomic variation identified in this study. The rarity of LGTs is somewhat surprising, as many studies have pointed out their importance during the evolution of the Mimiviridae (Iyer et al., 2006; Filee et al., 2007, 2008; Moreira and Brochier-Armanet, 2008; Yutin et al., 2013).

The Phycodnaviridae family displays clearly different patterns of genome evolution (Figure 3). *Ostreococcu*s viruses demonstrate a slight tendency of genome expansion for most of their representatives whereas *Chlorella* viruses display very different orientations including significant genome reduction for MA-1D and NYs-1, significant genome expansion for NY2A and relative stability for the others. A similar situation is observed for the three *Micromonas* virus genomes (Table 1). Another striking difference between the Phycodnaviridae and the Mimiviridae is the location of the genomic variations: there are scattered along the genomes for the Phycodnaviridae rather than being concentrated at the extremities as observed for the Mimiviridae. Interestingly, a study of laboratory variants of *Chlorella* viruses has evidenced the occurrence of large deletions at the left ends of the genomes (Landstein et al., 1995). However, an analysis of naturally occurring *Chlorella* viruses showed that insertion/deletion are more disseminated, corroborating our results (Nishida et al., 1999). Insertion and excision of mobile genetic elements represent a significant part of the *Chlorella* virus genomic mutations (23% of the total events). This observation confirms the importance of the GIY-YIG mobile endonuclease, associated or not with introns, and IS607 transposons during the genome evolution of the *Chlorella* viruses (Filee et al., 2007). *Chlorella* viruses also display an important number of multigene families gene losses/gene duplications. which account for 40% of the genomic events whereas they represent only 15–20% of the total gene content (Filee et al., 2008). Conversely, *Ostreococcus* virus and *Micromonas* viruses do not display any mobile elements movement and have a low amount of gene duplication (Figure 1, Table 1). Another difference is the importance of LGTs in *Ostreococcus* and *Micromonas* viruses. LGT is by far the major evolutionary force acting on the genomes of these viruses representing more than one third of the total number of mutations observed here. This observation contrasts with the rarity of LGT observed in the other GVs.

Taken together, these data suggest that there is no general trend of genome expansion or reduction in the family. Indeed, each group of viruses displays specific patterns of gene gain/gene loss. mimiviruses, megaviruses, and *Chlorella* viruses are mainly affected by gene loss and duplication targeting the multigene families. Multigene families appear as the main evolutionary tools in these viruses. *Ostreococcus* and *Micromonas* viruses are principally affected by LGTs and display few events of gene duplications.

### Characterization of the LGTs involved a large diversity of sources and protein functions

**Figure 4:**
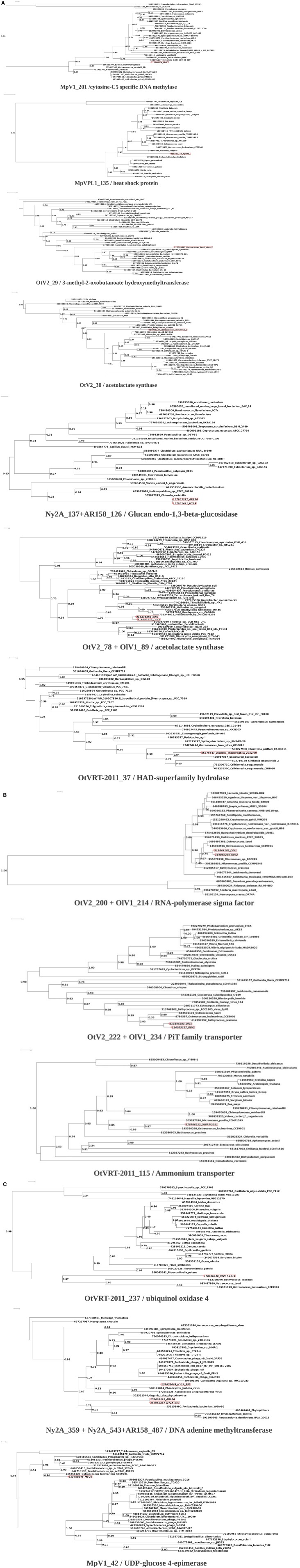
**Phylogenies of different cases of LGT encountered in GVs. (A)** LGT from diverse Bacteria and Eukarya **(B)** LGT from the hosts. **(C)** LGT from ambiguous sources. Viral sequences have been framed in red. The numbers beside each node indicate the value of the SH-like statistical test.

Genomic comparison of closely related GVs lead to the identification of 71 individual cases of gene transfers (Supplementary Table 1). Representative sets of phylogeny of these genes with identifiable functions are given Figure 4. As previously noticed, the vast majority of the LGTs concern the *Ostreococcus* and *Micromonas* viruses. Indeed, only 4 transfers for the *Chlorella* Phycodnaviruses and 5 for the mimiviruses and the megaviruses were observed. Most of these transfers concern genes coding proteins of unknown functions. Among the 24 genes displaying strong sequence similarity with genes associated with identifiable functions, 9 are homologs of DNA methyl transferase/methylase genes. These enzymes are components of Restriction-Modification systems, a prokaryotic system of defense against invading DNA. These enzymes are prone to LGTs and may behave as selfish mobile genetic elements (Kobayashi, 2001). The other functions implicated in LGTs compose a heterogeneous set of diverse informational or metabolic proteins: a Cytochrome B5, a Heat Shock protein, a RNA polymerase sigma factor etc. Interpretation of the phylogenies is generally unequivocal: GV genes are most often well nested among a forest of bacterial or eukaryotic genes (MpV-201, MpVPL1_135, OtV2_29, OtV2_30, NY2A_137, OtV2_78 + OlV1_89, OtVRT-2011_115) (Figure 4). Thus, this situation strongly favors a LGT polarized from the cell to the virus. Sometimes, the GVs sequences appear as a sister group of the host sequences, well nested inside the eukaryotic sub-tree (OtV2_200/OlV1_214, OtV2_222/OlV1_234, OtVRT-2011_115 etc…) (Figure 4). This pattern indicates strongly that the viruses have acquired the genes from their algal hosts. However, more ambiguous patterns are also encountered (Figure 4): in the OtVRT-2011_237 tree, the *Ostreococcus* viral sequence is located close to host sequence but distantly related from the other eukaryotic sequences (the tree has been rooted with Bacterial sequences). In this case, the polarization of the transfers is unclear. However, the fact that this gene is absent in all Phycodnaviruses, with the exception of OtVRT-2011, favors the hypothesis of a gene transfer from the cells to the virus. The NY2A_359/NY2A_543/AR158_487 gene phylogeny is even more puzzling: several Phycodnaviridae and Mimiviridae sequences are represented but form a deeply polyphyletic group. In this case, a possible explanation involves two independent LGTs from bacteria. Alternatively the sequence NY2A_359 has been acquired from another GVs, possibly a Mimiviridae infecting “Alga” as a *Phaeocystis* virus or an *Aureococcus* virus. Finally, in the MpV1_42 gene phylogeny, the viral sequence appears as a sister taxa with the *Ostreococcus lucimarinus* sequence (a potential host). However, the GV and host sequences are also closely related with diverse cyanobacterial sequences and their associated phages. This situation leads to two possible scenarios:

- The “two steps scenario” implies first, acquisition of the bacterial gene by MpV1 and second, transfers of the viral genes to the host genome.
- Alternatively, it could be envisaged independent captures of the bacterial gene by the virus and the alga.

The “two steps scenario” could provide an interesting situation in which the virus has served as a “shuttle” for an inter-kingdom LGT.

**Figure 5:**
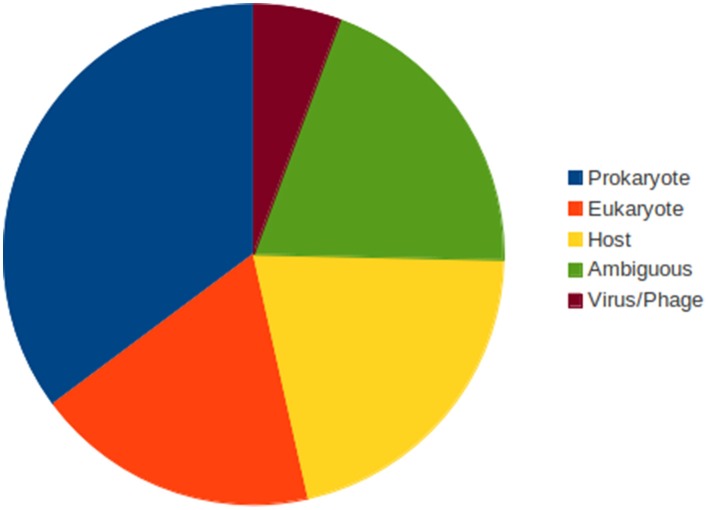
**Taxonomic sources of the LGTs**.

Prokaryotes represent the predominant source of genes acquired by LGTs (Figure 5). Various phyla are involved including Cyanobacteria, Proteobacteria, Firmicutes, Spirochetes, Verrucomicrobia… (Supplementary Table 1). Genes derived from the hosts and from other eukaryotic organisms represent additional but minor sources. These include mainly fungi and various marine organisms (*Hydra*, the anemone *Nematostella*, the crustacean *Daphnia*, the ascidian *Ciona*, diverse alga as *Chlorella* or *Cyanidioschyzon* etc…). Finally, 4 virus-to-virus LGTs were also identified, two implicating other GVs (a Marseillevirus and a Mimivirus), the two others ones with Cyanophages. Moreover, a substantial fraction of the LGTs displays ambiguous phylogenies blurring the identification of the donors (Figure 5). Possible additional cases of virus-to-virus LGTs are also possible (OtV2_40, OtVRT-2011_55, MpV1_243, MpVPL147…). As discussed previously with the NY2A_359/NY2A_543/AR158_487 gene phylogeny, the trees display complex evolutionary histories with possible multiple and independent acquisitions of the genes by the viruses rather than LGTs between the viruses themselves.

In summary, more than 70 cases of LGTs with a clear polarization from cells to the GVs have been evidenced. These transfers concern genes encoding proteins of very diverse functions and prokaryotes are the predominant source of gene acquisitions. LGTs from the hosts and from other eukaryotic organisms also occurred and isolated cases of gene transfers between viruses, including GV-to-GV LGTs, are also reported.

## Discussion

Comparative genome analysis of closely related GVs revealed that there are no general trends of genome reduction or genome expansion. Genomic mutations occur at a clock-like, constant rate, in both GV lineages but each group displays specific patterns of genome evolution:

- GVs with the largest genome as the Mimiviridae (mimiviruses and megaviruses) evolved mainly by a balanced process of duplications and losses of multigene families. Paralogous gene families appear as a genomic toolbox by providing most of the new genes but subsequent losses equilibrate the game and genome expansion is not discernible. LGTs and movements of mobile genetic elements are additional but marginal forces of genome evolution.
- Viruses with the smallest genomes as the *Ostreococcus* and *Micromonas* phycodnaviruses derive most of their genetic novelties by LGTs from a large variety of sources including various prokaryotic and eukaryotic species. Again, gene losses have balanced the process and globally the genomes appear with a relatively stable size.
- *Chlorella* phycodnaviruses which have intermediate genome sizes display more complex patterns of evolution, combining gene duplications and gene losses of multigene families in addition to numerous insertion/excision of IS607 Insertion Sequences and GIY-YIG mobile endonucleases. At the opposite, LGTs are rarely observed. These processes lead one of these *Chlorella* viruses to significant genome expansion, whereas the others display some genome reduction or genome size stability. It is noteworthy that the data support the fact that these mobile genetic elements are effectively able to transpose in the GV genomes. Their presences do not result from successive and independent LGTs from cellular sources. Abundance of these selfish genetic elements in GV genomes is a very specific feature among eukaryotic viruses (Filee et al., 2007). Indeed with the exception of the polydnaviruses that have a symbiotic, intra-cellular life style (Dupuy et al., 2011), mobile genetic elements are absent or very rare in eukaryotic viruses (Gilbert et al., 2014). Thus, the results presented here indicate that their importance during the evolutionary history of the GVs should not be underestimated.

It should be noted that the *Ostreococcus* and the *Micromonas* viruses are more distantly related from each others (80–90% identity of the four taxonomic markers with the ancestral sequence) than the Mimiviridae (97–99%) and the *Chlorella* viruses (94–99%). Despite their long period of divergence, the genome synteny of the *Ostreococcus* and the *Micromonas* viruses are still well conserved. By opposition, the genome co-linearity has been largely disrupted between the mimiviruses and the megaviruses: only a 600 kb central segment is conserved (Arslan et al., 2011; Yoosuf et al., 2012). Gene duplication and gene losses that occur at high rate at the extremities of the Mimiviridae genomes explain the fast disruption of the genome synteny. Similar processes are acting on the *Chlorella* virus genomes but the repartition of the genomic mutations is not biased toward the extremities. Among the 35 partial or nearly complete *Chlorella* virus genomes, this process leads to a fast and complete disappearance of the synteny, even for relatively close viruses (Jeanniard et al., 2013). Thus, the remarkable level of genome co-linearity of the *Micromonas* and *Ostreococcus* viruses despite a long period of divergence from their common ancestor might reflect fundamental different evolutionary processes compared to the GVs with larger genomes. Additionally, specific constraints on the genome size, for example for the packaging of the DNA into the capsid, might also limit the extent of genome variation and expansion. Interestingly, Mimiviridae and *Chlorella* Viruses display a large spectra of host specificity compared to *Ostreococcus* and *Micromonas* viruses. Mimiviruses have been isolated from Amoeba, but recent data indicate that they are also found in human (Kutikhin et al., 2014), leeche (Boughalmi et al., 2013), or hydra (Grasis et al., 2014). *Chlorella* phycodnaviruses are able to infect a large variety of protists harboring *Chlorella* symbiont (Van Etten, 2003) but can also infect mammals (Yolken et al., 2014). By contrast, *Ostreococcus* viruses display a strong host specificity (Clerissi et al., 2012). Thus, it is tempting to suggest that the broad host range of mimiviruses and *Chlorella* viruses are linked to their propensity to generate paralogous gene families, allowing the viruses to adapt to new hosts or changing ecological niches. In support to this hypothesis, some of the larger multigene families encode virus-host interaction functions (ankyrin-repeat, protein kinase, glysosyl transferase, FNIP-like repeats etc…). Interestingly this hypothesis offers further developments to determine the genes and molecular mechanisms which allow these GVs to adapt to a wide range of hosts, including diverse mammals, including human in relation with their potential health care concerns.

The paucity of LGTs detected in the genome of the Mimiviridae and the *Chlorella* viruses examined here is intriguing as many studies pointed out the importance of gene transfers during the evolution of these groups (Iyer et al., 2006; Filee et al., 2007, 2008; Moreira and Brochier-Armanet, 2008; Yutin et al., 2013). It was proposed that the sympatric lifestyle of these viruses with various prokaryotic species (symbionts or preys of their protist hosts) have facilitated the access to a very diverse gene pool as sources of new genes and functions (Filee et al., 2007; Raoult and Boyer, 2010). However, we identified only 4 LGTs for the *Chlorella* viruses and 5 for the Mimiviridae. This observation raises the question of the over-estimation of the importance of LGTs during the evolution of the GVs. An important point is the determination of the polarization of the transfers and the interpretation of the phylogenies. A large fraction of phylogenies including viral and cellular genes are inconclusive: low level of resolution, complex patterns involving multiple transfers/losses, ambiguities in the polarization as previously stated (Forterre, 2010). Interestingly, acquisition of numerous GV genes by various eukaryotes has been evidenced recently (Filee, 2014; Maumus et al., 2014). Thus, some LGTs wrongly polarized from the cell to the virus correspond in turn to acquisition of GV genes by cells. However, an important fraction of the GV genes possibly acquired from cells have homologs only in prokaryotes (Filee et al., 2007). In this case, the polarization of the transfers is unequivocal as it seems unlikely that prokaryotes frequently acquired genes from eukaryote viruses. Interestingly, the major part of LGTs identified in this study concern genes from bacterial origin suggesting that the possible over-estimation of the importance of LGTs essentially concerns genes from eukaryotic (host) origins. Finally, LGTs are the most frequent genomic events observed in the *Ostreococcus* and *Micromonas* viruses, but these viruses are distantly related to each other compared to the Mimiviridae and *Chlorella* viruses. This suggests that LGTs play a significant role in GV evolution, but they probably arise at a lower rate than previous thought for several GV lineages in which gene duplications/losses or movements of mobile genetic elements are the prevalent evolutionary forces. Additionally, it is also possible that many LGTs identified previously in the literature have occurred early, before the diversification of the different GV families. Supplementary data with a broader viral genome sampling and comparisons of more distantly GVs are clearly needed to address the exact importance of LGTs in the evolution of this viral family.

Taken together, these interpretations are not compatible with the hypothesis indicating that GVs derive from a cellular ancestor *via* progressive genome degradation in each lineage (Claverie, 2006). These data also reject the “pick pocket” hypothesis in which GVs experience massive LGTs leading to genome expansion (Moreira and Lopez-Garcia, 2005). By contrast, these data support the idea of a “genomic accordion” in which GVs evolved through a balanced process of gene gains (duplication mainly but also LGTs) compensated by gene losses. Indeed, each GV lineage most likely has a specific pattern of genome evolution, probably reflecting different host-virus interactions. However, there is little doubt that the last common ancestor of the Megavirales was a relatively simple virus, encoding a limited genomic repertoire that has successively expanded over the times (Yutin et al., 2014; Krupovic and Koonin, 2015). Our results indicate that the rate of genome expansion has slow down before the diversification of the Mimiviridae and the *Chlorella* viruses. Thus, these data support the idea that the ancestor of these viral families was already a GV, indicating that genome gigantism has emerged early during the evolution of the Megavirales (Yutin et al., 2009).

### Conflict of interest statement

The author declares that the research was conducted in the absence of any commercial or financial relationships that could be construed as a potential conflict of interest.
