# Supplementary material for: Genomic comparison of closely related Giant Viruses supports an accordion-like model of evolution
Source: Front Microbiol. 2015 Jun 16;6:593. doi: 10.3389/fmicb.2015.00593 (PMC4468942; doi:10.3389/fmicb.2015.00593)
Supplement: Supplementary file 1 [file Table1.PDF]

Supplementary Table 1: List of LGTs identified in this study

ORF name	Function	First BLAST hit	E value	Source of the gene transfer
OtV1_8	?	Ajellomyces dermatitidis	0.0005	Eukaryote
OtV1_33/OsV5_33	?	Coccomyxa subellipsoidea C-169	4E-014	Eukaryote
OtV1_113/OsV5_131	?	Nematostella vectensis	1E-039	Eukaryote
OtV1_160	?	Micromonas pusilla CCMP1545	0,0008	Eukaryote
OtV1_171	phytanoyl-CoA dioxygenase	Methylovulum miyakonense	0,0002	Bacteria
OsV5_124	DNA methyl transferase	Phaeocystis globosa virus	1E-087	Ambiguous
OsV5_125	?	Organic Lake phycodnavirus	1E-039	Virus
OsV5_159	?	Pseudoalteromonas luteoviolacea	1E-032	Bacteria
OsV5_218	?	Cyanidioschyzon merolae strain 10D	2E-035	Eukaryote
OtV2_3	DNA-cytosine methyltransferase	Brachyspira murdochii	5E-105	Bacteria
OtV2_4	?	Geminocystis herdmanii	0,0003	Bacteria
OtV2_29	3-methyl-2-oxobutanoate hydroxymethyltransferase	Acetothermia bacterium	8E-081	Bacteria
OtV2_30	acetolactate synthase	Pelagibacter ubique	3E-113	Bacteria
OtV2_40	?	Synechococcus phage S-SSM4	1E-017	Ambiguous
OtV2_78/OlV1_89	DNA methylase	Helicobacter sp. MIT 05-5293]	5E-094	Bacteria
OtV2_158/OlV1_172	?	Daphnia pulex	0,000000004	Eukaryote
OtV2_165	?	Synechocystis sp. PCC 7509	8E-013	Bacteria
OtV2_167	phosphogluconate dehydrogenase	Acanthamoeba castellanii str. Neff	1E-057	Ambiguous
OtV2_179	?	Prochlorococcus phage Syn1	0,00003	Virus
OtV2_193/OlV1_206	?	Hydra vulgaris	5E-010	Eukaryote
OtV2_201/OlV1_213	Cytochrome B5	Ostreococcus lucimarinus CCE9901	1E-035	Host
OtV2_202/OlV1_214	RNA-polymerase sigma factor	Ostreococcus tauri	0,000000001	Ambiguous
OtV2_222/OlV1_234	PiT family transporter	Ostreococcus lucimarinus CCE9901	0.0	Host
OlV1_4	serine/threonine-protein kinase	Fibrisoma limi BUZ 3	9E-029	Bacteria
OlV1_7	?	Micromonas sp. RCC299	8E-078	Host
OlV1_12	?	Ostreococcus lucimarinus CCE9901	6E-040	Host
OlV1_190	Nucleotide-rhamnose synthase/epimerase-reductase	Populus trichocarpa	9E-037	Eukaryote
OlV1_236	?	Micromonas sp. RCC299	8E-016	Host
OtVRT-2011_6	?	Chlorella variabilis	1E-018	Eukarya
OtVRT-2011_37	HAD-superfamily hydrolase	uncultured bacterium	3E-025	Bacteria
OtVRT-2011_38	?	Pleurocapsa sp. PCC 7319	0,00000001	Bacteria
OtVRT-2011_55	?	Acanthamoeba polyphaga mimivirus	3E-028	Ambiguous
OtVRT-2011_115	Ammonium transporter	Ostreococcus tauri	0.0	Host
OtVRT-2011_149	?	Fusobacterium sp. CAG:439	0,00001	Bacteria
OtVRT-2011_169	?	Burkholderia sp. UYPR1.413	1E-044	Bacteria
OtVRT-2011_183	?	Acinetobacter nectaris	3E-047	Bacteria
OtVRT-2011_225	?	Micromonas pusilla virus PL1	3E-056	Host
OtVRT-2011_233	ubiquinol oxidase 4	Micromonas sp. RCC299	8E-085	Host
OtVRT-2011_242	?	Paenibacillus sp. JCM 10914	0,0003	Bacteria
OtVRT-2011_244	?	Ciona intestinalis	1E-033	Eukarya
MpV1_8	?	Ostreococcus tauri virus RT-2011	3E-020	Host
MpV1_9	?	Micromonas sp. RCC299	8E-070	Host
MpV1_42	UDP-glucose 4-epimerase	Ostreococcus lucimarinus CCE9901]	4E-102	Ambiguous
MpV1_114	?	Micromonas pusilla CCMP1545	0,000000001	Host
MpV1_201	cytosine-C5 specific DNA methylase	Rickettsia bellii	2E-078	Bacteria
MpV1_203	?	Insectomime virus	1E-064	Virus
MpV1_225	?	Flavobacterium psychrophilum	0,0007	Bacteria
MpV1_243	?	Bathycoccus sp. RCC1105 virus BpV2	1E-045	Ambiguous
MpVPL1_24/MpVSP1_156	?	Micromonas pusilla CCMP1545	0,000000007	Host
MpVPL1_26	?	Grosmannia clavigera kw1407	0,00006	Eukaryote
MpVPL1_45	?	Magnetospirillum sp. SO-1	9E-051	Bacteria
MpVPL1_47	DNA methylase	Phaeocystis globosa virus	2E-142	Ambiguous
MpVPL1_70/MpVSP1_102	?	Bathycoccus prasinos	1E-154	Host
MpVPL1_89/MpVSP1_178	?	Toxoplasma gondii GAB2-2007	0,00003	Eukaryote
MpVPL1_135	heat shock protein	Micromonas pusilla CCMP1545	0.0	Host
MpVPL1_167	DNA methyltransferase	Verrucomicrobia bacterium SCGC AAA164-N20	2E-124	Ambiguous
MpVPL1_191	?	Enhygromyxa salina	4E-040	Eukaryote
MpVSP1_175	?	Diaphorina citri	0,000000006	Eukaryote
MpVSP1_211	FkbM family methyltransferase	Desulfovibrio africanus	3E-012	Bacteria
MpVSP1_212	?	uncultured bacterium	4E-042	Bacteria
MpVSP1_228	?	Rhodospirillum rubrum	3E-011	Bacteria
MpVSP1_242	?	Micromonas sp. RCC299	3E-055	Host
Ny2A_137/AR158_126	Glucan endo-1,3-beta-glucosidase	Helicosporidium sp. ATCC 50920	6E-052	Ambiguous
Ny2A_359	DNA adenine methyltransferase	Sphingopyxis alaskensis RB2256	9E-049	Ambiguous
Ny2A_543/AR158_487	DNA adenine methyltransferase	Cafeteria roenbergensis virus BV-PW1	6E-051	Ambiguous
Ny2A_542/AR158_486	?	Synechococcus phage S-SSM5	2E-012	Virus

Terra2_822702_824003	?	Candidatus Amoebophilus asiaticus	1E-029	Bacteria
Terra2_859909_854765	?	Phaeocystis globosa virus	1E-040	Ambiguous
Terra2_1152701_1153423	?	Pseudomonas sp. WCS374	5E-056	Bacteria
Chiliensis_647	?	Acanthamoeba polyphaga moumouvirus	0.0	Ambiguous
Courdo11_298	?	Bacillus pumilus	2E-080	Bacteria
